# Study of the anti-cancer activity of a mesoporous silica nanoparticle surface coated with polydopamine loaded with umbelliprenin

**DOI:** 10.1038/s41598-024-62409-0

**Published:** 2024-05-20

**Authors:** Sedigheh Edalatian Tavakoli, Alireza Motavalizadehkakhky, Masoud Homayouni Tabrizi, Jamshid Mehrzad, Rahele Zhiani

**Affiliations:** 1 Department of Biochemistry, Neyshabur Branch, Islamic Azad University, Neyshabur, Iran; 2 Department of Chemistry, Neyshabur Branch, Islamic Azad University, Neyshabur, Iran; 3 Department of Biology, Mashhad Branch, Islamic Azad University, Mashhad, Iran

**Keywords:** Umbelliprenin, Mesoporous silica nanoparticles, Polydopamine, Drug delivery, Cancer, Biochemistry, Biotechnology, Cancer

## Abstract

A mesoporous silica nanoparticle (MSN) coated with polydopamine (PDA) and loaded with umbelliprenin (UMB) was prepared and evaluated for its anti-cancer properties in this study. Then UMB-MSN-PDA was characterized by dynamic light scattering (DLS), Field emission scanning electron microscopy (FESEM), Transmission electron microscopy (TEM) and FTIR methods. UV-visible spectrometry was employed to study the percentage of encapsulation efficiency (EE%). UMB-MSN-PDA mediated cell cytotoxicity and their ability to induce programmed cell death were evaluated by MTT, real-time qPCR, flow cytometry, and AO/PI double staining methods. The size of UMB-MSN-PDA was 196.7 with a size distribution of 0.21 and a surface charge of −41.07 mV. The EE% was 91.92%. FESEM and TEM showed the spherical morphology of the UMB-MSN-PDA. FTIR also indicated the successful interaction of the UMB and MSN and PDA coating. The release study showed an initial 20% release during the first 24 h of the study and less than 40% during 168 h. The lower cytotoxicity of the UMB-MSN-PDA against HFF normal cells compared to MCF-7 carcinoma cells suggested the safety of formulation on normal cells and tissues. The induction of apoptosis in MCF-7 cells was indicated by the upregulation of P53, caspase 8, and caspase 9 genes, enhanced Sub-G1 phase cells, and the AO/PI fluorescent staining. As a result of these studies, it may be feasible to conduct preclinical studies shortly to evaluate the formulation for its potential use in cancer treatment.

## Introduction

Cancer chemotherapy still suffers from significant drawbacks due to the side effects of anticancer drugs^1^. New nano drug delivery systems (NDDSs) have gained increasing attention in recent years to reduce the adverse effects associated with these drugs^2^. In addition to nanotubes and liposomes, inorganic nanoparticles and dendrimers are some of the NDDSs^3–6^. A class of NDDS called mesoporous silica nanoparticles (MSNs) has received considerable attention from many researchers as a result of their convenient scalability, tunable pore size, and a large pore volume and surface area^7^. As a result of these properties, MSNs can encapsulate and deliver drugs rapidly and are easy to prepare, such as through sol-gel methods in aqueous solutions^8^. Several MSN-based nanoplatforms have been developed for drug delivery since the Food and Drug Administration deemed silica-based materials safe for use^9^. To enhance MSN tumor targeting properties, some researchers have used active targeting groups, while others have relied on the tumor microenvironment, such as low pH and higher glutathione levels. Chemically responsive MSNs have been designed to release chemotherapeutic drugs in response to pH and/or redox^9–11^.

It is important to coat the surface of nanoparticles with organic polymers in order to control their properties since nanoparticles interact with their surroundings. For instance, nanoparticles' colloidal stability depends critically on their coatings, including a coating that inhibits aggregation at various pH values or in salty environments. In addition, the coating has the potential to be further functionalized, such as by adding targeting agents, biomolecules, imaging probes, and drugs^12^. The self-polymerization of dopamine in basic environments results in a layer of polydopamine (PDA) adhering strongly to nanoparticle surfaces without any prior treatment^13^. The concentration and time of dopamine reaction can easily be adjusted to control the diameter of the PDA layer. Furthermore, Catechol/quinone groups can be used to further functionalize the PDA layer^14^. The surface modification of PDA was easy, and PEGylation improved its dispersibility and stealth. By enhancing the permeation and retention (EPR) effect, nanoparticles can be conjugated, expanding their applications in therapy, visualization, and sensing^15^.

Several secondary metabolites have been isolated from natural sources, including plants, in recent years, and there is an outstanding level of interest in the potential biomedical usage of these compounds^16,17^. There are, however, a considerable number of these bioactive compounds that are insoluble in biological fluids, and different chemical or physical treatments should be performed to increase their bioavailability^18^. As a highly insoluble and bioactive sesquiterpene coumarin with many biological properties, umbelliprenin (UMB) was specifically selected because of its hepatoprotection, gelatinase inhibition, anticancer properties, antigenotoxicity, and antileishmanial properties^19–22^. The results of the new research have shown that UMB inhibits the activity of matrix metalloproteases and oxidosqualene cyclases, and it also exhibits pro-apoptotic properties and anticancer effects as well^23,24^. Additionally, UMB has been demonstrated to increase lymphocytes’ responses to mitogens and induce immunologic responses in tumor microenvironment. Furthermore, this compound may also affect angiogenesis and decrease metastasis as a result of its intervention with fibrinolytic system^25^.

This study aimed to synthesize the UMB-loaded MSN that was decorated with PDA (UMB-MSN-PDA). The UMB-MSN-PDA was characterized using dynamic light scattering (DLS), field emission scanning electron microscopy (FESEM) and Fourier transform infrared (FTIR). Then the in vitro cytotoxic properties and pro-apoptotic activities of UMB-MSN-PDA were evaluated by MTT assay, flow cytometry, and real-time qPCR.

## Materials and methods

### Materials

UMB, ammonium fluoride (NH4F), tetraethyl orthosilicate (TEOS), cetyltrimethylammonium bromide (CTAB), propidium iodide (PI), acridine orange (AO), 3-(4,5-dimethylthiazol- 2-yl)- 2,5-diphenylte-trazolium bromide (MTT) and PDA were supplied by Sigma-Aldrich (Darmstadt, Germany). Cell culture medium, antibiotics, and fetal bovine serum (FBS) were purchased from Gibco (United States).

### Preparation of the UMB-loaded MSNs (UMB-MSNs)

In distilled water (DW), CTAB (5 mM) and NH4F (80 mM) are dissolved (50 mL final volume) in a stirring system and heated to 80 ℃ for 6 h. The solution is then added to 10 mL of TEOS (1 mg/mL) and incubated for 20 min at 80 ℃. After washing the nanoparticles three times with ethanol and DW and centrifuged (12000, rpm, 10 min), the solvent was removed using a rotary evaporator (Heidolph, Germany) at 40 ℃. Next, to remove CTAB, the obtained powder was dissolved in 40 ml of ethanol containing 80 µl of 37% hydrochloric acid and refluxed at 80 ℃ for 24 h, then the sample was centrifuged and refluxed again for another 24 h. It is repeated and centrifuged at the end, and after washing several times at a temperature of 40 ℃, it is dried under vacuum.

To add the drug to the nanoparticles, a certain amount of MSN is dissolved in an aqueous solution containing the drug in 10 mg dissolved in 5 ml of distilled water and placed on a stirrer, and incubated at room temperature for 24 h. Then it is centrifuged, washed, and dried at 40 ℃ under a vacuum using a rotary evaporator.

### UMB-MSN surface coating with PDA (UMB-MSN-PDA)

Dopamine (12 g) was added to 25 mg UMB-MSN dispersed in 12 mL Tris buffer (pH 8.0) and incubated for 6 h in the dark. After that, the sample was centrifuged (12000 rpm, 10 min) three times to wash and freeze-dried (24 h).

### Characterization

Nanoparticles were dispersed in 10 mL of DW and measured by DLS (Nano-ZS, Malvern, UK) for size and zeta potential. In order to do DLS analysis, the 50 µL of nanoparticle suspension was added to 950 µL of DW. For evaluating the zeta potential 50 µL of nanoparticles suspension were mixed with 950 µL 10 mM NaCl. The functional groups of UMB-MSN-PDA were determined by FTIR analysis (AVATAR 370, Thermo Nicolet, USA) after they were compressed into tablets with KBr powder. A grid was sprayed with dispersed nanoparticles in DW to determine their morphology. Nanoparticles were analyzed using FESEM after drying and coating.

### Drug entrapment

The amount of unencapsulated UMB was determined using UV spectroscopy, where the amount of free UMB in the supernatant was measured. Knowing the total amount of UMB, the amount of encapsulated was determined by subtracting the amount of unencapsulated from the total amount. To check for drug entrapment, a UV-vis spectrophotometer was used to measure at 326 nm. A standard graph of the absorption was drawn using serial dilution of free UMB. Based on the formula below, the amount of unencapsulated UMB and the encapsulation efficiency (EE%) of the formulation were determined:

$$    {\text{EE }}\left( \%  \right) = \left( {{\text{total amount of drug}} - {\text{free drug}}} \right)/{\text{total amount of drug }} \times \left( {100} \right)   $$

### Release of UMB form formulation

The free UMB and UMB release from UMB-MSN-PDA was investigated using UV spectroscopy. Dialysis tubing was immersed in PBS with pH 7.4, pH 6.5, and pH 5.5 stirred at 37 ℃, while free UMB and UMB-MSN-PDA were poured into the bags (12–14 kDa MWCO). At the defined time intervals (6, 12, 24, 48, 72, 96, 120, 144, and 168 h) 1 mL of the sample was drawn and replaced by 1 mL of fresh medium. To analyze the solution, a release curve was drawn using the UMB standard curve, and the concentration of the UMB was determined at each time, and cumulative release was calculated.

### Cytotoxic assay

MCF-7 human breast cancer and HFF human foreskin fibroblast cell lines were obtained from the Pasteur Institute of Iran, Tehran, Iran. The cells were seeded on 96-well plates (5 × 10^3^ cells per well) and incubated overnight at 37 ℃. Then they were treated with various concentrations of nanoparticles and free UMB for 48 h. Then the media were removed and MTT dye was added per each well. After 240 min of incubation in the dark, the formazan crystals were dissolved by adding DMSO. These samples were then analyzed with the ELISA Reader method at 570 nm to determine their absorbance. The following formula was used to calculate cell viability:

$$ {\text{Cell viability}}(\% ) = ({\text{OD treatedcells}}/{\text{OD untreatedcells}}) \times (100) $$

### Apoptosis assay using flow cytometry

MCF-7 cells were seeded in 6-well plates (10^6^ cells per well) and incubated overnight at 37 ℃. Following treatment with nanoparticles for 48 h, the medium was drawn and cells detached to examine cell cycle and apoptosis. Incubation of the sediment with PI dye after centrifugation (1500 rpm, 5 min) was followed by flow cytometry analysis using the FACSCalibur (BD, USA).

### Fluorescent microscopy

10^6^ cells were seeded in each well of a 6-well plate and incubated overnight at 37 ℃. Following treatment with different concentrations of UMB-MSN-PDA, the cells were washed, trypsinized, and centrifuged for 5 min at 1500 rpm. At ambient temperature, AO and PI were added to the cell and incubated for 10 min in the dark. An inverted fluorescence microscope (Eclipse TE2000-U, Nikon, Japan) was used to examine the cells.

### Real-time qPCR

In real-time qPCR, primers are provided for measuring the expression of p53, caspase-8, and caspase-9 in Table 1. Different concentrations of UMB-MSN-PDA were used for 48 h on the cells. A Biofact RNA extraction kit (South Korea) was used to extract total RNA. In the next step, RNA was evaluated using a nanodrop, cDNA was synthesized, and real-time qPCR was performed with the real-time PCR (CFX96, Bio-Rad, USA).

**Table 1:** List of primers for real-time PCR.

Gene	Forward 5′-3′	Reverse 5′-3′
P53	TCAGATCCTAGCGTCGAGCCC	GGGTGTGGAATCAACCCACAG
Caspase 8	GAAAAGCAAACCTCGGGGATAC	CCAAGTGTGTTCCATTCCTGTC
Caspase 9	CCAGAGATTCGCAAACCAGAGG	GAGCACCGACATCACCAAATCC
GAPDH	TGCTGGTGCTGAGTATGTCG	GCATGTCAGATCCACAACGG

### Statistics

The findings were investigated using GraphPad Prism (Version 8) and significant differences were considered at p < 0.05. All the experiments were performed in three-time repeats. All data were expressed as mean value ± SD.

### Ethics approval and consent to participate

All institutional and national guidelines for the care and use of laboratory animals were followed.

## Results

### UMB-MSN-PDA characterization

**Figure 1:**
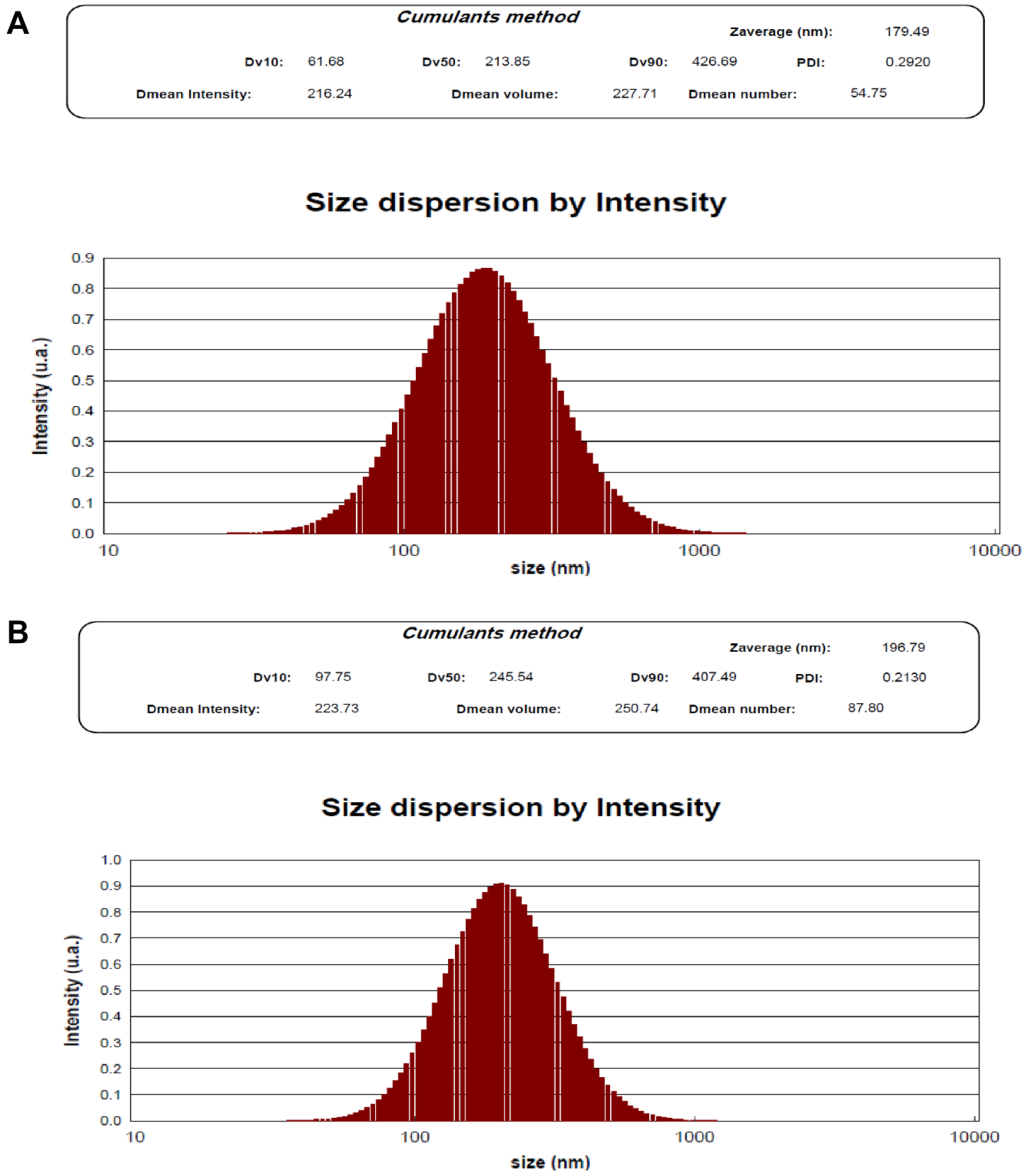
Nanoparticle characterization. The results of size were reported by DLS (**A**) UMB-MSN and (**B**) UMB-MSN-PDA.

**Figure 2:**
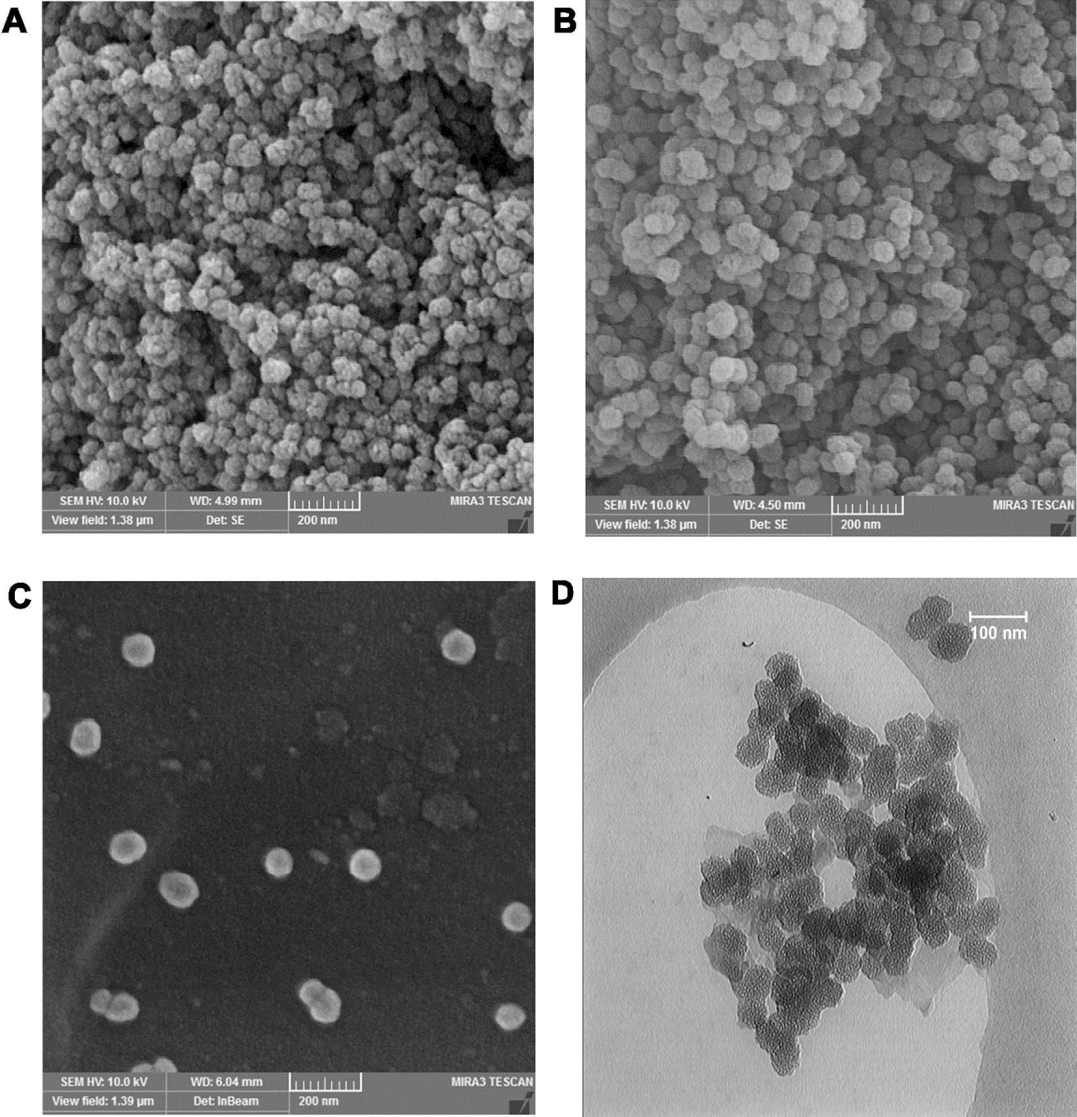
Nanoparticle characterization. The FESEM micrograph of the (**A**) blank MSN, (**B**) UMB-MSN, and (**C**) UMB-MSN-PDA. (**D**) The TEM micrograph of the UMB-MSN-PDA.

**Figure 3:**
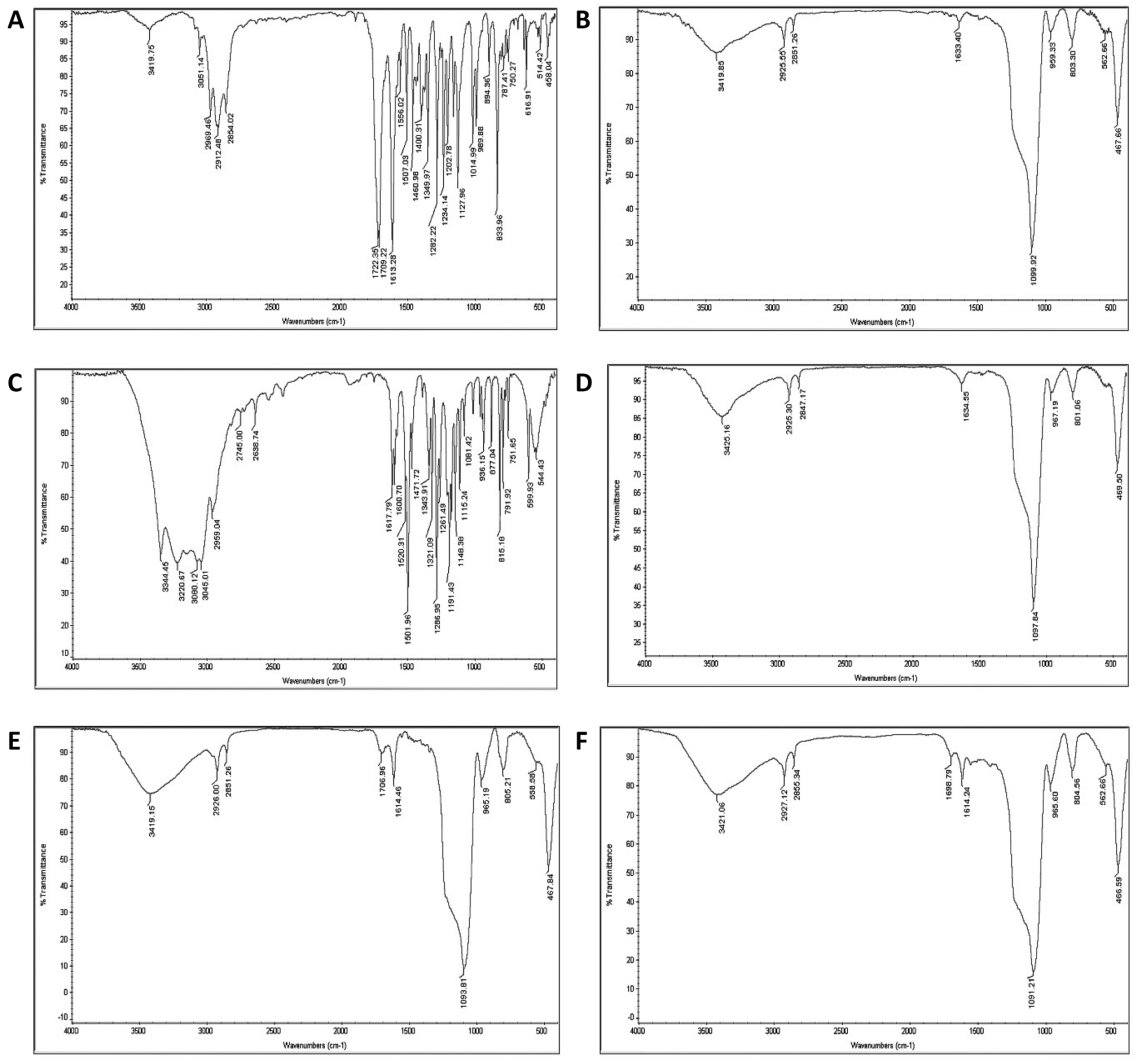
FTIR assay. FTIR spectra of the (**A**) free UMB, (**B**) Blank MSN, (**C**) dopamine, (**D**) Blank MSN-PDA, (**E**) UMB-MSN and (**F**) UMB-MSN-PDA.

Figure 1A and B show the hydrodynamic diameter of the UMB-MSN before and after coating with PDA using DLS. As demonstrated in Fig. 1A the Z-average of the UMB-MSN was 179.49 nm with a polydispersity index (PDI) of 0.29 and a mean number of 54.75 nm. Figure 1B shows the Z-average of UMB-MSN-PDA was 196.79 with a PDI of 0.21 and a mean number of 87.80 nm. The zeta-potential of the UMB-MSN was -30.04 ± 7.44 and the surface charge of the UMB-MSN-PDA was −41.07 ± 7.45. Figure 2A–C demonstrates the FESEM micrograph which indicated the spherical morphology of the blank MSN, UMB-MSN, and UMB-MSN-PDA and a size range consistent with the mean number as reported by DLS. In addition, the increase in the size of MSN was indicated after coating with PDA. Figure 2D, demonstrates the TEM micrograph of the UMB-MSN-PDA, in which mesopore structures is indicated. Figure 3A–F demonstrates the FTIR results of the free UMB, Blank MSN, dopamine, Blank MSN-PDA, UMB-MSN, and UMB-MSN-PDA, respectively. To evaluate UMB-MSN and PDA-MSN interactions successfully in the final formulation, all compounds were tested for compatibility with FTIR spectroscopy. UMB FTIR spectrum indicated several characteristic peaks at 1613.28 cm^−1^, 1709.22 cm^−1^, 1722.35 cm^−1^, 2854.02 cm^−1^, and 2912.48 cm^−1^ (see Fig. 3A). These peaks could be observed in Fig. 3E and F which demonstrated the UMB-MSN and UMB-MSN-PDA respectively and resulted in the presence of the UMB in the formulation. The presence of dopamine in the formulation was demonstrated by the peak 1617.79 in the Fig. 3C which corresponds to the peaks 1634.55 and 1698.89 in Fig. 3C and F in which the PDA was surface—decorated on blank MSN and UMB-MSN. The EE% was calculated at 91.92% using UV spectrometry.

### Release study

**Figure 4:**
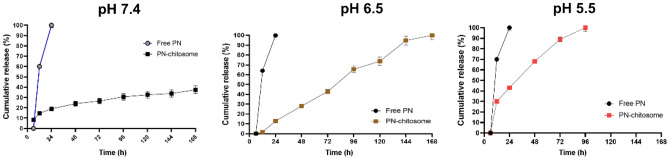
Release study. Cumulative release of the free UMB and UMB from UMB-MSN-PDA in various conditions including pH 7.4, pH 6.5, and pH 5.5 during 168 h.

Figure 4 demonstrates the release of free UMB and UMB from UMB-MSN-PDA formulation in various pH conditions. Initially, there was a sudden release of 20% over the first 24 h and kept a plateau until 168 h. During 168 h of the release study less than 40% of the drug was released from the UMB-MSN-PDA. In pH 6.5 the release rate was more rapid and 100 of UMB was released from formulations during 168 h. In pH 5.5 100% of UMB release from formulation was reached during 96 h.

### Cytotoxicity study

**Figure 5:**
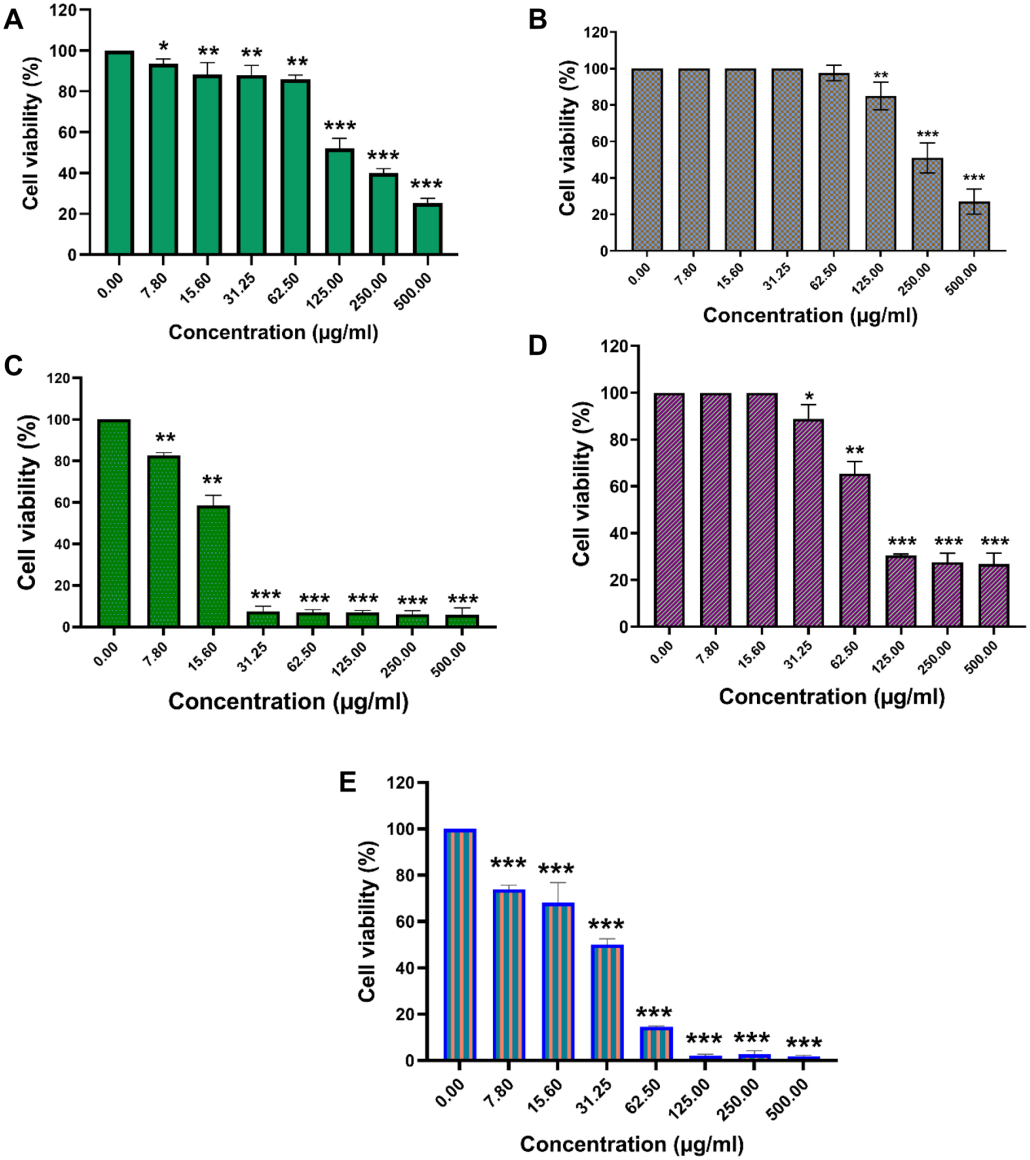
Cytotoxicity assay. (**A**) The cell viability diagrams of the HFF normal cells treated with various concentrations of free UMB, (**B**) The cell viability diagrams of the HFF normal cells treated with various concentrations of UMB-MSN-PDA, (**C**) Cell viability diagram of the MCF-7 cells treated with various concentrations of UMB, (**D**) Cell viability diagram of the MCF-7 cells treated with various concentrations of blank MSN-PDA, and (**E**) Cell viability diagram of the MCF-7 cells treated with various concentrations of UMB-MSN-PDA. (*p < 0.05, **p < 0.01, and ***p < 0.001). The data are presented as mean ± SD. The test was performed in triplicate.

HFF and MCF-7 were used to test the cytotoxic effects of UMB and UMB-MSN-PDA. Figure 5A and B show the cytotoxic results of free UMB and formulation against HFF cells, in which in the high concentrations (125, 250, and 500 μg/mL) the cytotoxic effects were significant in comparison with cells without treatment (control group) (**p < 0.01 and ***p < 0.001). In comparison to the formulation, free UMB showed higher levels of cytotoxicity against HFF cells. The cytotoxicity of Free UMB, as demonstrated in Fig. 5C, against MCF-7 cells was significantly high and started with concentrations of 7.8 µg/mL (**p < 0.01 and ***p < 0.001). Figure 5D and E show the cytotoxicity of Blank-MSN-PDA and UMB-MSN-PDA on the MCF-7 cells. The highest statistically significant cytotoxicity was found in UMB-MSN-PDA at all doses compared to the negative control (***p < 0.001). The cytotoxic effect of the Blank-MSN-PDA was less than both Free UMB and -UMB-MSN-PDA, and it was statistically significant at the dose of 31.25 µg/mL and above (*p < 0.05, **p < 0.01, and ***p < 0.001).

### Evaluation of the apoptosis

**Figure 6:**
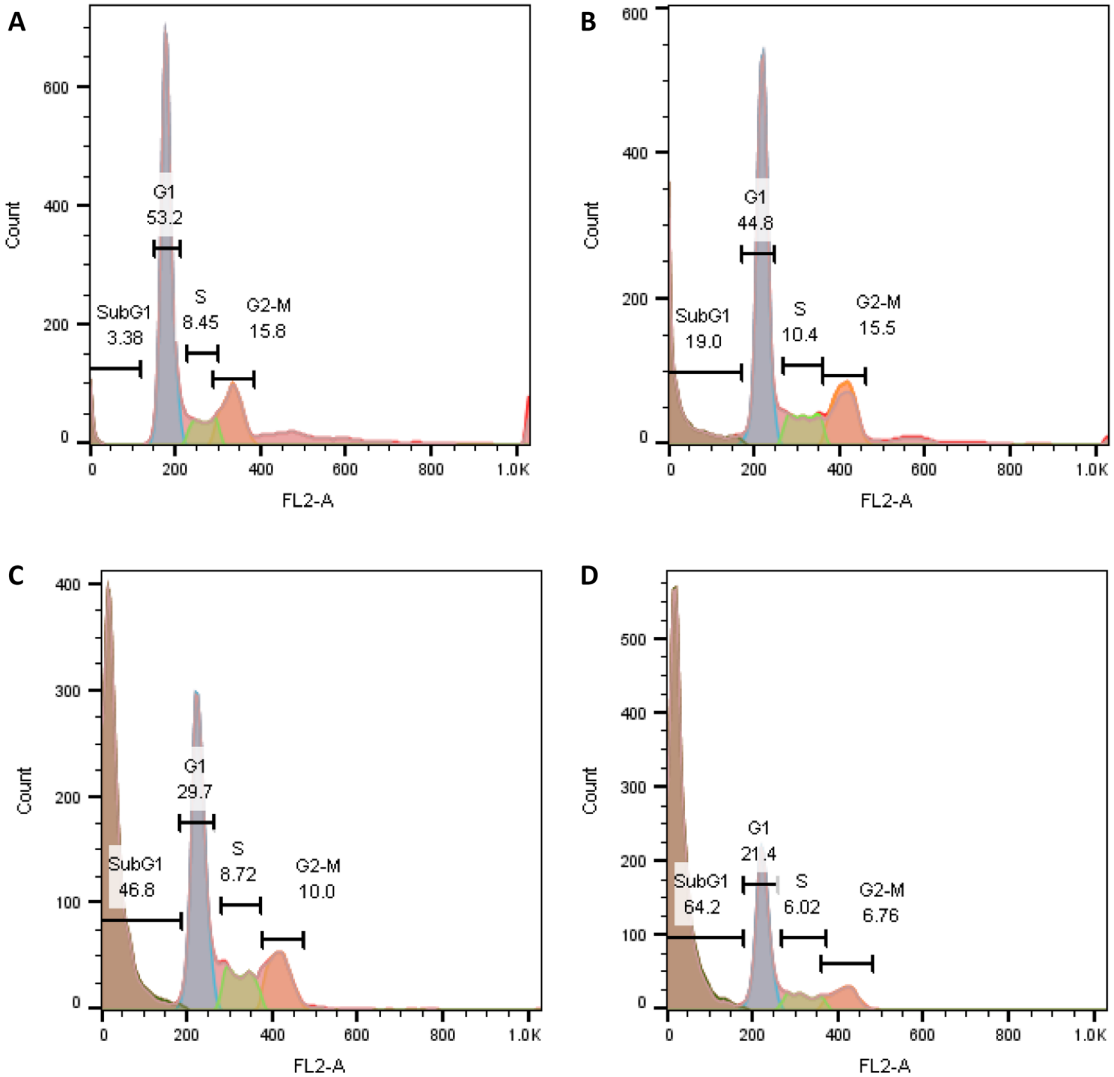
Apoptotic effects of UMB-MSN-PDA. Effects of different concentrations (**A**) control, (**B**) 15 (**C**) 30, and (**D**) 45 µg/mL of UMB-MSN-PDA on MCF-7 cells.

UMB-MSN-PDA’s impacts on the cell cycle were studied using flow cytometry. With increasing concentrations, UMB-MSN-PDA causes the stop at the SubG1 phase (Fig. 6). As it is demonstrated, only 3.3% of the cells in the control sample were in SubG1. Compared to the treated samples, 19, 46.8, and 64.2% of the cells were arrested in the SubG1 phase when UMB-MSN-PDA was used at concentrations of 15, 30, and 45 µg/ml. Apoptosis was induced in cells treated with UMB-MSN-PDA.

**Figure 7:**
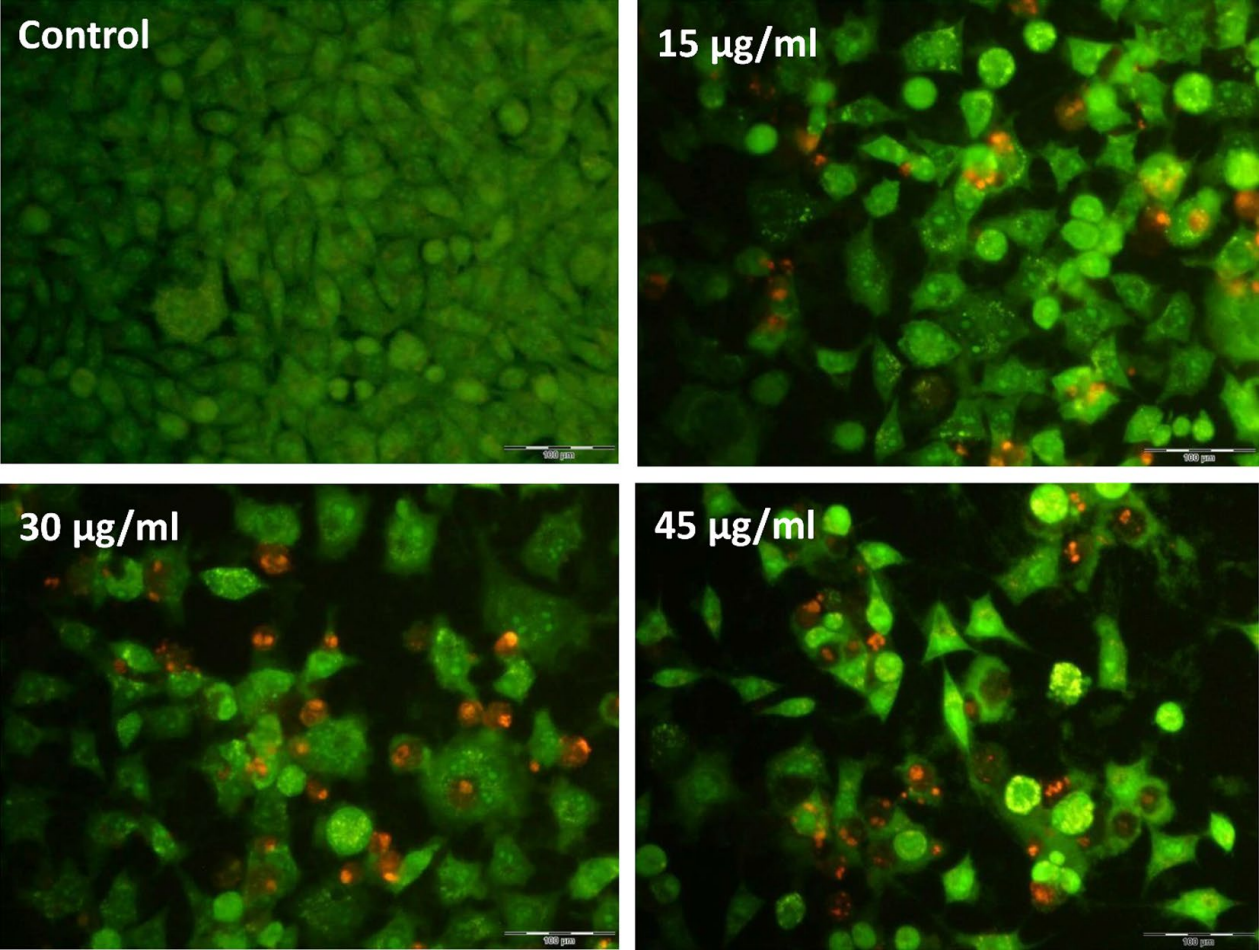
Fluorescent microscopy. Effects of various concentrations control, 15, 30, and 45 µg/mL of UMB-MSN-PDA on MCF-7 cells using AO/PI double staining.

A cytotoxic and apoptotic effect of UMB-MSN-PDA at various concentrations is shown by AO and PI analysis (Fig. 7). In the control group, no morphological changes have occurred in control cells, as their nucleus and cytoplasm emit uniform green fluorescence. As UMB concentration increases and apoptosis is more likely to occur, the PI dye penetrates damaged cells. Apoptosis increases with increasing concentration of treatment.

### Real-time qPCR

**Figure 8:**
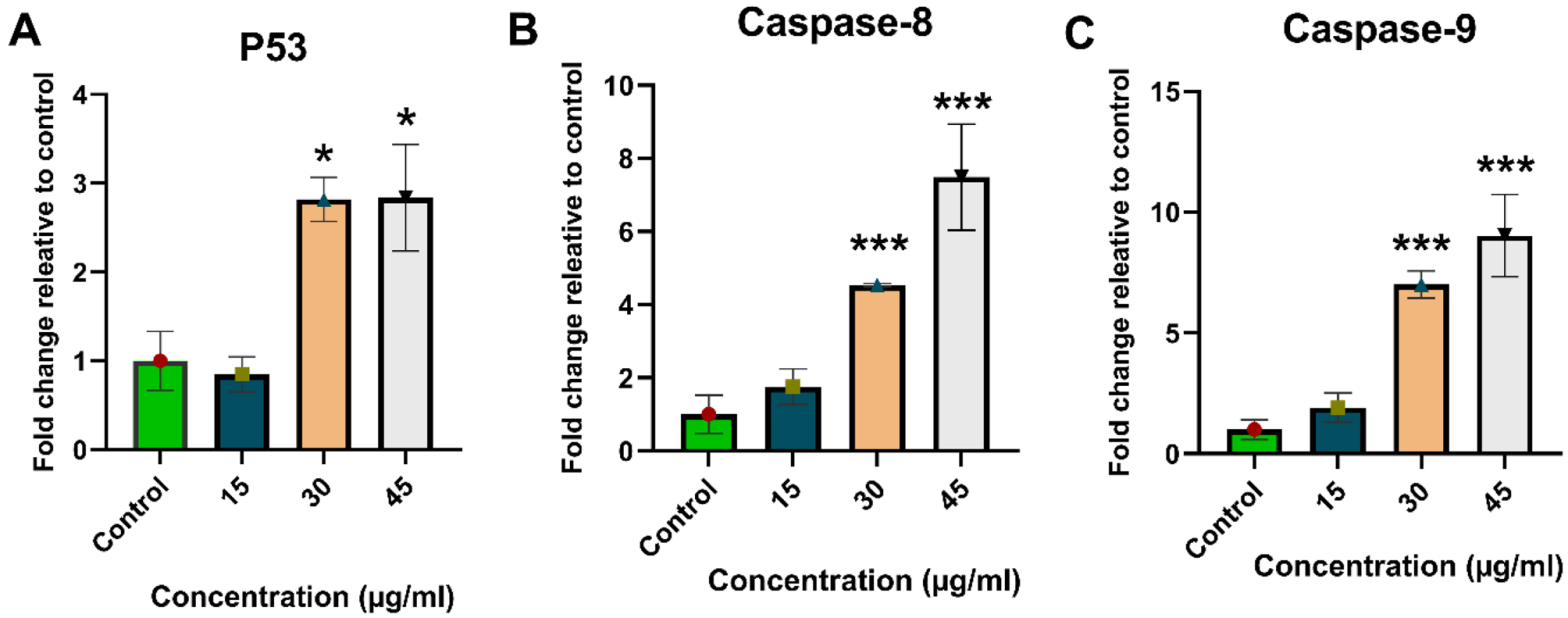
Results of real-time qPCR. Quantitative analysis of P53, Caspase 8, and Caspase 9 genes expression as apoptosis-related factors in cells treated with UMB-MSN-PDA. (*p < 0.05, and ***p < 0.001). The data are presented as mean ± SD. The test was performed in triplicate.

A change in gene expression has been observed over time for the investigated genes. The activation of the apoptotic pathway within the cells can be demonstrated by this observation (Fig. 8). It has been shown that UMB-MSN-PDA increases the expression levels of P53, caspase 8, and caspase 9 in apoptotic cells. At the concentrations of 30 and 45 ug/mL an almost threefold significant increase was observed in the P53 gene compared with the control group (*p < 0.05). In the case of caspase 8, this increase was fourfold and sevenfold in concentrations of 30 and 45 ug/mL, respectively, which were statistically significant (***p < 0.001) when compared to the control group. Finally, there was a sevenfold and ninefold increase in caspase 9 gene expression at the doses of 30 and 45 µg/mL. These increases were also significant (***p < 0.001).

## Discussion

The purpose of the current study was to prepare UMB-MSN-PDA and improve their therapeutic efficacy against cancer. A hydrodynamic size of 196.7 nm was found for the UMB-MSN-PDA in this study. It would be advantageous to have particles that are smaller than 200 nm to penetrate tumor tissue, which consists of an irregular endothelium^26^. MSN and PDA shells can be used to implement unique NDDS applications^27^. In terms of dispersibility and stealth, the PDA was not very effective. PEG or other chemicals were used to modify the surface of the material in order to enhance its dispersibility and stealth properties. As a consequence of the EPR effect, tumor accumulation was also observed. As nanoparticles are highly scalable, PDA-coated nanoparticles can be used for different applications in therapy, imaging, and sensing^28^. PDA coatings are generally negatively charged as a result of reverse dissociation and protonation/deprotonation. The zeta potential value, however, can vary significantly depending on the measurement conditions^29–31^. Zheng and colleagues. Have developed an NDDS for controlling drug release by coating drug-loaded MSN with PDA. By using PDA as a gatekeeper, this system inhibits drug release. In acidic environments, such as those found in endosomes, PDA disassembles, releasing drugs slowly^32^. Using PDA-coated MSN functionalized with tocopherol polyethylene glycol succinate (TPGS), Cheng et al. demonstrated a doxorubicin (DOX)-loaded nanocarrier system using nanoparticles loaded with DOX. In vivo, MSNs-DOX-PDA-TPGS exhibits an ability to conquer multidrug resistance, compared to DOX and DOX-loaded nanoparticles without the TPGS ligand modifications^33^. In the present study, after surface decoration with PDA, UMB-MSN nanoparticles changed size from 179.4 nm to 196.7 nm, indicating successful surface modification^34^. Here, according to SEM data, UMB-MSN-PDA has spherical morphology and DLS data confirms nanoparticle uniformity. The TEM data indicated the mesopore structures.

The UMB was also examined using FTIR spectroscopy. An infrared spectrum of UMB showed many characteristic peaks that had disappeared. The amides peak at 1556 cm^−1^, the carbonyl peak at 1722 cm^−1^ and the amino peak at 3051 cm^−1^ of UMB diminished, as well as the stretching vibration of the benzene ring skeleton at 1507 cm^−1^. Due to FTIR’s ability to detect only infrared absorption by surfaces, UMB has been encapsulated after loading^35^. The absence of a new peak indicates that there has been no interaction between UMB and MSN, and the encapsulation process has not altered the structure of UMB^36^.

A controlled pattern of drug release was obtained using PDA nanoparticles in a pH-responsive manner^37^. By controlling the release of drugs, cancer treatment strategies could be more effective^38^. Chen et al. used polydopamine-coated MSNs to control drug release in response to pH stimuli. Over 40% of the drug was released when pH 6.5 was reached, while only 25.63% was released at pH 7.4^39^. Under acidic conditions, polydopamine degrades, leading to controlled release. A tumor’s microenvironment becomes acidic when lactic acid builds up during cell division. Warburg effect describes this phenomenon. It is suggested that by exposing UMB-MSN-PDA to an acidic environment, pH-sensitive nanoparticles with pH-sensitive properties are activated, causing their detachment of PDA and UMB release^40^.

A cytotoxicity test was conducted on UMB-MSN-PDA against cancer and normal cells. At the concentrations tested, the formulations showed cytotoxicity against cancer cells, while UMB-MSN-PDA exhibited only minimal cytotoxicity against normal cells when compared to MCF-7 cancer cells. The higher levels of metabolism could be due to the higher cytotoxicity of the formulation which suggested that this formulation of UMB in MSN could protect normal cells and tissues against adverse effects of UMB administration, hence may reduce the side effects^41–43^. As a result of mutations and abnormal behaviors, cancer cells grow and divide uncontrollably and have higher metabolism. This makes them more vulnerable to nanoparticle cytotoxicity than normal cells. In addition to increasing toxicity in cancer cells, while sparing normal cells, nanoparticles can target specific cellular pathways and structures, increasing toxicity in cancer cells^44,45^.

Cancer cells treated with UMB-MSN-PDA showed strong dose-dependent G1 arrest. The flow cytometry study found that UMB reduced the number of S-phase cells and raised the number of G1-phase cells that were arrested. According to our analysis, nanoparticles decreased G1, S, and G2/M phase cell percentages and enhanced sub-G1 phase cells. In addition, UMB-MSN-PDA induced apoptosis in a dose-dependent manner based on fluorescent microscopy data. Several factors contribute to apoptosis, including membrane permeation, condensation of chromatin, and shrinkage of cells. In the apoptotic process, fluorescence staining can distinguish the early and late phases^46^. In vitro staining methods, such as AO/PI double staining, are used to analyze cell morphological characteristics. As AO binds to different organelles of cells, it emits different colors of fluorescence. Upon binding of AO to ssDNA rather than dsDNA, it emits an orange fluorescence color. Upon binding to dsDNA, AO emits green fluorescence. Due to its ability to penetrate early apoptotic cell membranes, AO may be able to detect fragments of DNA in those cells. As PI cannot pass the membrane of a living cell, it can only be indicated in a dead cell. A necrotic cell or a late apoptotic cell can be detected by red fluorescence emission^47^.

UMB-MSN-PDA treated cells showed enhanced levels of gene P53 expression, followed by caspase 8, and caspase 9, indicating that nanoparticles could promote apoptosis. Several studies have shown that caspase-8 is also associated with cell metastasis, angiogenesis, and other cancer-promoting effects^48^. Researchers have discovered that caspase-8 is an important p53 target gene that is induced by cytotoxic drugs. Caspase-8-mediated p53/p73-dependent apoptosis was induced by etoposide in HNSCC cells by caspase-8-mediated positive feedback amplification^49^. It has been suggested that increased intracellular ROS levels, change in the permeability of the mitochondrial membrane, the cytochrome C release, and subsequent activity of the apoptotic pathway may all contribute to UMB-MSN-PDA inducing apoptosis^50^. The P53 plays an essential function in the signaling process of apoptosis. At the transcriptional level, this protein can activate caspase signaling and induce apoptotic pathways in cancer cells by enhancing and reducing pro-apoptotic and anti-apoptotic genes, respectively^51,52^. By stopping the cell cycle, the P53 protein prevents cancer cells from proliferating^53^. After apoptosis signals are received, caspase 3, -6, -7, -8, and -9 are activated, cleaving apoptosis-related proteins^54^. Apoptosis and the production of cytokines are both regulated by caspase-8, a cysteine protease. Upon recruitment into a multimeric complex, caspase-8 becomes active through autoactivation or trans-cleavage by another caspase. Active Caspase-8 propagates apoptosis signals by cleaving BH3 Bcl2-interacting proteins or by cleaving downstream caspases^55^. It was found that UMB-MSN-PDA promotes the expression levels of activated P53, caspase 8, and caspase 9, leading to the induction of cell apoptosis.

## Conclusion

UMB-MSNs synthesized have a size of 179.4 nm and uniform distribution. PDA layers were applied to their surfaces, resulting in 196.7 nm in size. UMB-MSN-PDA exhibited a high inhibitory effect against cancer cells in the study. The results of this study suggest nanoparticles may not have adverse effects on normal cells or tissues as they do not cause any toxicity. Our findings indicated that UMB-MSN-PDAs lead to enhanced expression levels of the P53, caspase 8, and caspase 9. The substantial apoptotic pathway activation in MCF-7 cells resulted in the suppression of these cells when treated with UMB-MSN-PDA. It has also been demonstrated that UMB-MSN-PDA has proapoptotic effects by changing gene profiles related to cell death. These data were confirmed by a rise in the number of cells in the SubG1 phase using flow cytometry, and AO/PI double staining obtained by fluorescence microscopy. As a result of these studies, it may be feasible to conduct preclinical studies in the near future to evaluate the formulation for its potential use in cancer treatment.

## Data Availability

The datasets used and/or analyzed during the current study are available from the corresponding author upon reasonable request.
